# Adiponectin ameliorates lung ischemia–reperfusion injury through SIRT1-PINK1 signaling-mediated mitophagy in type 2 diabetic rats

**DOI:** 10.1186/s12931-021-01855-0

**Published:** 2021-10-03

**Authors:** Tao Jiang, Tianhua Liu, Xijin Deng, Wengang Ding, Ziyong Yue, Wanchao Yang, Xiangqi Lv, Wenzhi Li

**Affiliations:** Department of Anesthesiology (Hei Long Jiang Province Key Lab of Research On Anesthesiology and Critical Care Medicine), The Second Affiliated Hospital, Harbin Medical University, No.194, XueFu Road, NanGang District, Harbin, China

**Keywords:** Adiponectin, SIRT1, Mitophagy, Lung ischemia–reperfusion injury, Type 2 diabetes mellitus

## Abstract

**Background:**

Diabetes mellitus (DM) is a key contributing factor to poor survival in lung transplantation recipients. Mitochondrial dysfunction is recognized as a critical mediator in the pathogenesis of diabetic lung ischemia–reperfusion (IR) injury. The protective effects of adiponectin have been demonstrated in our previous study, but the underlying mechanism remains unclear. Here we demonstrated an important role of mitophagy in the protective effect of adiponectin during diabetic lung IR injury.

**Methods:**

High-fat diet-fed streptozotocin-induced type 2 diabetic rats were exposed to adiponectin with or without administration of the SIRT1 inhibitor EX527 following lung transplantation. To determine the mechanisms underlying the action of adiponectin, rat pulmonary microvascular endothelial cells were transfected with SIRT1 small-interfering RNA or PINK1 small-interfering RNA and then subjected to in vitro diabetic lung IR injury.

**Results:**

Mitophagy was impaired in diabetic lungs subjected to IR injury, which was accompanied by increased oxidative stress, inflammation, apoptosis, and mitochondrial dysfunction. Adiponectin induced mitophagy and attenuated subsequent diabetic lung IR injury by improving lung functional recovery, suppressing oxidative damage, diminishing inflammation, decreasing cell apoptosis, and preserving mitochondrial function. However, either administration of 3-methyladenine (3-MA), an autophagy antagonist or knockdown of PINK1 reduced the protective action of adiponectin. Furthermore, we demonstrated that APN affected PINK1 stabilization via the SIRT1 signaling pathway, and knockdown of SIRT1 suppressed PINK1 expression and compromised the protective effect of adiponectin.

**Conclusion:**

These data demonstrated that adiponectin attenuated reperfusion-induced oxidative stress, inflammation, apoptosis and mitochondrial dysfunction via activation of SIRT1- PINK1 signaling-mediated mitophagy in diabetic lung IR injury.

## Introduction

Lung transplantation remains definitive therapy for end-stage respiratory failure with no other treatment options [1]. However, approximately 15% of recipients undergoing lung transplantation experience graft complications due to lung ischemia reperfusion (IR) injury [2]. We and others have made enormous efforts to explore rescue strategies for lung IR injury in lung transplantation [3–5]. However, lung IR injury is still the major risk factor for postoperative complications, such as acute graft rejection and obliterative bronchiolitis [6]. Data from the International Society of Heart and Lung Transplantation Registry show that diabetes mellitus (DM) is an independent risk factor for mortality at both 1 and 5 years after lung transplantation, and an unexpectedly high prevalence of undiagnosed DM has been identified among patients awaiting lung transplantation [7]. Lung transplantation recipients may already be at an increased risk of DM prior to transplantation [8]. For example, patients with chronic obstructive lung disease (COPD) may already be taking glucocorticoids. Extensive efforts have been dedicated to exploring the rescue strategies for lung IR injury in the diabetic state. Our previous study showed that DM aggravated lung IR injury, and mitochondrial dysfunction was a pivotal factor in this process [9]. Thus, preservation of mitochondrial function is significant in the management of patients with pretransplant DM who undergo lung transplantation.

Mitochondrial homeostasis can be regulated through several mechanisms. Moderation and elimination of reactive oxygen species (ROS), and mitochondrial autophagy (mitophagy) have all been demonstrated to be critical mediators of mitochondrial homeostasis [10]. Mitophagy is crucial for mitochondrial quality control, which selectively removes dysfunctional or damaged mitochondria via autophagy, thereby preventing excessive reactive oxygen species (ROS) production and release of mitochondrial proapoptotic factors [11]. The role of mitophagy in IR injury remains controversial, and both adaptive and detrimental effects have been reported [12, 13]. Mitochondrial dysfunction has been suggested to be evident in diabetic complications due to decreased levels of mitophagy, leading to accumulation of damaged mitochondria and exacerbating tissue injuries [14]. Furthermore, whether mitophagy occurs in diabetic lung IR injury remains unclear. We believe that a better understanding of how mitophagy is regulated during diabetic lung IR injury, which is essential to developing a new therapeutic strategy to attenuate pulmonary dysfunction in diabetic lung transplantation recipients.

Silent information regulator 1 (SIRT1) is a nicotinamide adenine dinucleotide (NAD +)-dependent deacetylase that mediates a protective effect against IR injury, possibly through maintenance of mitochondrial function [15]. Of note, SIRT1 has been proposed to induce mitophagy contributing to the attenuation of mitochondrial dysfunction and subsequent IR injury [16]. We have previously demonstrated that normalizing or activating lung SIRT1 signaling conferred a pulmonary protective effect [17]. The question of whether lung SIRT1 may be a master regulator of mitophagy in diabetic lung IR injury represented a logical extension of the above studies.

Adiponectin (APN), an adipocytokine with a collagenous domain and a C-terminal globular domain and that is predominantly secreted by adipocytes, can modulate insulin responsiveness, and maintain mitochondrial homeostasis [18]. Plasma adiponectin levels are decreased in patients with insulin resistance, obesity, and type 2 diabetes [19]. Restoration of diabetes-induced hypoadiponectinemia by adiponectin is correlated with a decreased of IR insult and favorable functional recovery after IR injury [20]. Previous studies from our lab have indicated that adiponectin protectes against lung IR injury in type 2 diabetic rats, but the potential mechanisms remain poorly defined [21]. Accordingly, the aims of this study was to determine whether adiponectin treatment ameliorated diabetic lung IR injury by regulating mitophagy to protect against mitochondrial dysfunction through activation of the SIRT1 signaling pathway.

## Materials and methods

### In vivo study

#### Animals

Pathogen-free male Sprague–Dawley rats weighing 200–250 g at 8 weeks of age were obtained from the animal care facility of Harbin Medical University. All animal experiments were approved by the Institutional Animal Care and Use Committee at Harbin Medical University. All animal experiments were performed under the Guidelines on National Institutes of Health Guidelines for the Care and Use of Laboratory Animals (NIH Publication No. 85-23, revised, 1996). The animal care and experimental protocols were approved by the Ethical Board of the Second Affiliated Hospital of Harbin Medical University (Harbin, China; No. SYDW2020-022).

#### Establishment of a type 2 diabetic rat model

High-fat diet-fed streptozotocin-induced type 2 diabetic rat model was induced as described previously [9, 17]. Briefly, rats were fed with high-fat food containing (2.5% cholesterol, 5% sesame oil, 15% lard, 20% sucrose, and 57.5% normal chow) for 6 weeks followed by intraperitoneal injection of streptozotocin (35 mg/kg). After that, rats were continuously fed with high-fat food. The rats with fasting plasma glucose above 11.1 mmol/L 72 h after STZ injection were considered as diabetic. Next, we tested the glucose tolerance of various groups by conducting the intraperitoneal glucose tolerance test (IPGTT) and oral glucose tolerance test (OGTT) to confirm the successful establishment of the type 2 diabetic rat model. The standard laboratory chow-fed rats were studied as the nondiabetic controls.

#### Lung transplantation

Orthotopic left lung transplantation using the cuff technique procedure was carried out as described previously [3, 4]. Briefly, donor rats were anesthetized with sodium pentobarbital (30 mg/kg) administered intraperitoneally, intubated with 12-gauge catheter and ventilated with 40% oxygen (balance nitrogen) at a tidal volume of 10 ml/kg with 2 cm H_2_O positive end-expiratory pressure (PEEP). After heparinization, the donor left lung was flushed with 20 mL of low-potassium dextran solution at 4 °C at a perfusion pressure of 20 cm H_2_O through the pulmonary artery. The left lung was clipped and attached to a cuff tube, and preserved at 4 °C in the perfusion solution for 2 h.

The recipient rats were anesthetized and ventilated in the same manner as the donor rats. The right femoral artery was catheterized for pressure monitoring (Datex, Helsinki, Finland) and arterial blood sampling analysis (Bayer, Medfield, MA). After a left thoracotomy, the left pulmonary arteries, bronchi and left pulmonary veins were conjugated between donors and recipients by the cuff technique. During the lung transplantation, the tidal volumes were regulated to 6 mL/kg and restored to 10 mL/kg immediately after reperfusion. The recipients were extubated after recovery from anesthesia. The recipient rats were treated with 0.125% ropivacaine by local infiltration analgesia every 12 h. The recipients using type 2 diabetic rats were studied as diabetic lung transplantation. All rats were positioned on a heating pad to maintain body temperature and sodium pentobarbital was used to maintain anesthesia. The body temperature of individual rats was measured by a rectal thermometer and maintained between 37 °C and 39 °C. At 24 h after reperfusion, all rats were killed and lung grafts were collected.

#### Experimental groups

The study was divided into two parts. The first part was to explore mitophagy in diabetic lung IR injury, which included following groups: sham group (Con + Sham), lung IR group (Con + IR), DM + sham group (DM + Sham), DM + lung IR group (DM + IR). The second part was to determine the effect and mechanism of APN treatment on lung IR injury in type 2 diabetic setting, which included following groups: DM + lung IR group (DM + IR), DM + IR + adiponectin -treated group (DM + IR + A), DM + IR + adiponectin and EX527 (the inhibitor of SIRT1 signaling) treated group (DM + IR + A + S), and DM + IR + adiponectin + 3-methyladenine (3-MA) (autophagy inhibitor) treated group (DM + IR + A + M). Adiponectin (100 μg/kg, dissolved in 1.0 ml of sterile normal saline) was injected intravenously immediately after reperfusion following the lung transplantation. 3-methyladenine (15 mg/kg, intraperitoneally) was injected 30 min before the operative model of lung transplantation [22]. EX527 (5 mg/kg/day, intraperitoneally) was injected for 3 days before the surgery and once 20 min before the reperfusion as described previously [17]. The doses of adiponectin, EX527 and 3-MA were selected as described previously [17, 23].

#### Histological analysis

The lung tissues were fixed in paraformaldehyde and embedded in paraffin. 5-μm thickness sections were prepared and stained with hematoxylin and eosin. The degree of the lung injury was assessed for airway epithelial cell damage, neutrophil infiltration, hemorrhage, interstitial edema, and hyaline membrane formation, each criterion was scored on a semi-quantitative scale of 0–4 as follow: normal = 0, minimal change = 1, mild change = 2, moderate change = 3, and severe change = 4 [24, 25]. The degree of the lung injury was assessed in a blinded manner by 2 pathologists who were blinded to the study in 5 high-power fields that were randomly selected from each section.

#### Enzyme-linked immunosorbent assay

Serum concentrations of interleukin-6 and tumor necrosis factor-α were measured by enzyme-linked immunosorbent assay kits (R&D Systems, Minnesota, USA) according to the manufacturer’s protocols.

#### Terminal deoxynucleotidyl transferase dUTP nick end labeling (TUNEL) assay

Lung parenchymal cell apoptosis was detected by TUNEL using an In Situ Cell Death Detection kit (Roche Molecular Biochemicals, Mannheim, Germany) according to the manufacturer’s protocols. The TUNEL-positive cells that showed red nuclear staining and all of the cells with DAPI staining were counted within five randomly chosen fields in a blinded manner. The apoptosis index was calculated as the ratio of the number of apoptotic nuclei to the total number of nuclei counted. The microscopic images were then analyzed by Image J software to automatically detect the number of apoptotic nuclei and the total number of nuclei cells [4, 12, 26, 27].

#### Determination of MDA and SOD

Malondialdehyde (MDA) levels and superoxide dismutase (SOD) activity in lung tissues were measured by spectrophotometer using commercial kits (Jiancheng Bio-Technology, Nanjing, China) as previously described [9, 17].

#### Western blot analysis

Western blot analysis was carried out as described previously [9, 17]. Briefly, the lung tissue protein or cellular protein was separated by 12% sodium dodecyl sulphate–polyacrylamide gel electrophoresis and then transferred onto polyvinyl difluoride membranes. The membrane was blocked with 5% fat-free dry milk in tris-buffer solution Tween, the blots were incubated with primary antibodies SIRT1 (Cell Signaling Technology, 9475), Parkin (Cell Signaling Technology, 2132), LC3-II (Cell Signaling Technology, 3868), PINK1 (Abcam, ab186303), Acetyl-FoxO1 (Thermo Fisher Scientific, PA5104560) at 4 °C overnight. Then, the membranes exposed to the corresponding horseradish peroxidase-conjugated secondary antibody for 2 h. The membranes were visualized with diaminobenzidine staining and exposed to film. VDAC (Cell Signaling Technology, 4661) and GADPH (Cell Signaling Technology, 5174) were used as the internal control for loading variations. Band intensities were analyzed with Image J software.

#### Determination of SIRT1 activity

SIRT1 activity was evaluated using a fluorometric assay (SIRT1 fluorogenic Assay Kit, BPS Bioscience, San Diego, CA) as described previously[17].

#### Measurement of mitochondrial membrane potential

Mitochondrial membrane potential was assessed by a JC-1 staining kit (Sigma Aldrich, St. Louis, MO) as described previously [9]. Red fluorescence in healthy mitochondria is due to the potential-dependent formation of JC-1 aggregates (590 nm), while green fluorescent JC-1 monomers (530 nm) are detected in depolarized mitochondria. The fluorescence was detected by a fluorimeter (Infinite M200PRO, TECAN). Mitochondrial membrane potential was calculated as the ratio of aggregated JC-1 to monomeric JC-1.

### In vitro study

#### The culture and identification of rat pulmonary microvascular endothelial cells (PMVECs)

Rat PMVECs were isolated using the method described by Chen et al. [28] and modified by Li et al. [29] using the “tissue” method as previously described [30]. Briefly, the rats were euthanized by exsanguination, and the lungs were removed by sterile techniques. The visceral pleura was removed and peripheral lung tissue was cut into small pieces (< 1 mm^3^) in medium M199 containing 20% fetal bovine serum and 50 μg/mL endothelial cell growth supplement. Then, the fragments were placed in a 25 cm^2^ culture flask upside down adding penicillin–streptomycin (100 U/mL) in a 5% CO_2_, at 37 °C. After 60 h of culture, the tissues were removed and M199 was changed. PMVECs were identified according to the results of immunocytochemistry staining of CD31 and lectin binding.

#### Simulated IR

In vitro ischemia–reperfusion was performed as previously described [30]. PMVECs were placed in a sealed incubator and pre-ventilated with 95% O_2_ and 5% CO_2_ for 2 h.

##### Simulated cold storage

The sealed incubator was placed in 4 °C, and M199 culture medium was immediately replaced with low-potassium dextran solution (pH 7.2–7.4) with gas insufflation stoppage.

##### Simulated implantation

After 2 h of simulated cold storage, the incubators were kept at room temperature and sealed for 1 h to simulate the transplantation period.

##### Simulated reperfusion

Following the replacement of low-potassium dextran solution immediately with 37 °C preheated M199 culture medium (pH 7.2–7.4), the incubator was ventilated with 40% O_2_, 5% CO_2_ and 45% N_2_ for 24 h.

For simulated type 2 diabetic reperfusion, high glucose-high fat (HG/HF) M199 containing 15 mM glucose and saturated FFA palmitate (16:0; 500 μM) was used to simulate pathophysiology condition of diabetic state, while normal M199 culture medium was used as a control [31]. For adiponectin pretreatment, adiponectin (2 μg/mL) was used immediately after reperfusion [32]. Gas concentrations in the incubator were monitored with a gas analyzer (S/N 32,590; Datex Ohmeda, Helsinki, Finland).

#### Short interfering RNA and transfection

Short interfering RNA (siRNA) oligonucleotides against *SIRT1*, *PINK1* and their negative control siRNA were designed and synthesized by Gene Pharma (Shanghai, China). The sequences of siRNA oligonucleotides were as follows: *SIRT1* siRNA, 5′-CAUUGUUAUUGGGUCUUCUCUGAAATT-3′ (sense) and 5′- UUUCAGAGAAGACCCAAUAACAAUGTT-3′ (antisense), *PINK1* siRNA, 5′-GCAGCGUAGCAUGUCUGAUUUTT-3′ (sense) and 5′-AAAUCAGACAUGCUACGCUGCTT-3′ (antisense). PMVECs were transfected with siRNA using Lipofectamine 2000 (Invitrogen, Rockford, USA) as previously described [9].

#### Immunofluorescent staining

PMVECs were fixed with 4% paraformaldehyde and permeabilized with 0.2% Triton X-100, blocked with 5% bovine serum albumin, and subsequently incubated with primary antibodies. After washing with PBS, cells were exposure to secondary antibodies conjugated to an Alexa fluorophore. Fluorescent images were obtained with a laser scanning confocal microscope. Five randomly selected fields from one coverslip were included to calculate an average, and experiments were repeated independently at least 3 times.

#### Cell viability

The effect of high glucose on the cell viability for PMVECs was evaluated by performing WST-8 assay using Cell Counting Kit-8 (CCK8, Dojindo Laboratories, Japan), and the absorbance was assessed at 450 nm using a microplate reader (Bio‑Rad iMark; Bio‑Rad Laboratories, Hercules, CA, USA).

#### Apoptosis assay

Annexin V-7-AAD apoptosis assay kit (Biotech, China) was used to measure apoptosis of PMVECs by flow cytometry following the manufacturer’s instructions.

#### Mitochondrial membrane potential and mitochondrial ROS

Mitochondrial membrane potential was measured by exposing PMVECs to JC-1 molecular probes (Invitrogen, Calif, USA) following the manufacturer’s instructions. Mitochondrial membrane potential was expressed as the ratio of red to green fluorescence areas. To assess mitochondrial ROS production, cells were incubated with MitoSOX (Life Technologies, USA). Mitochondrial ROS generation was visualized by fluorescence microscopy.

#### Measurement of mitochondrial morphology

The mitochondrial disruption was evaluated by the Flameng score [33] as follows: 0, structures are normal and particles are intact; 1, structures are normal and particles are lost; 2, mitochondria are swollen but matrices are clear; 3, cristae are broken and matrices are concentrated; 4, cristae are extensively destroyed and the membranes are ruptured.

### Statistical analysis

Wet/dry weight ratio, arterial blood gas analysis, the apoptosis index, the lung injury score, and Flameng scores are expressed as medians (interquartile range) and were compared by the Kruskal–Wallis test, and the *P* value was adjusted with Dunn’s. The remaining data are expressed as the mean ± standard deviation (SD) and were compared using ANOVA followed by Bonferroni post hoc test or using a 2-tailed Student *t* test for unpaired observations. Statistical testing was examined using Prism software package version 5.0 (GraphPad Software, La Jolla, CA, USA). A value of *P* < 0.05 was considered to indicate a statistically significant difference. In vitro experiments were repeated at least 3 times.

## Results

### Characterization of diabetic animals

**Fig. 1:**
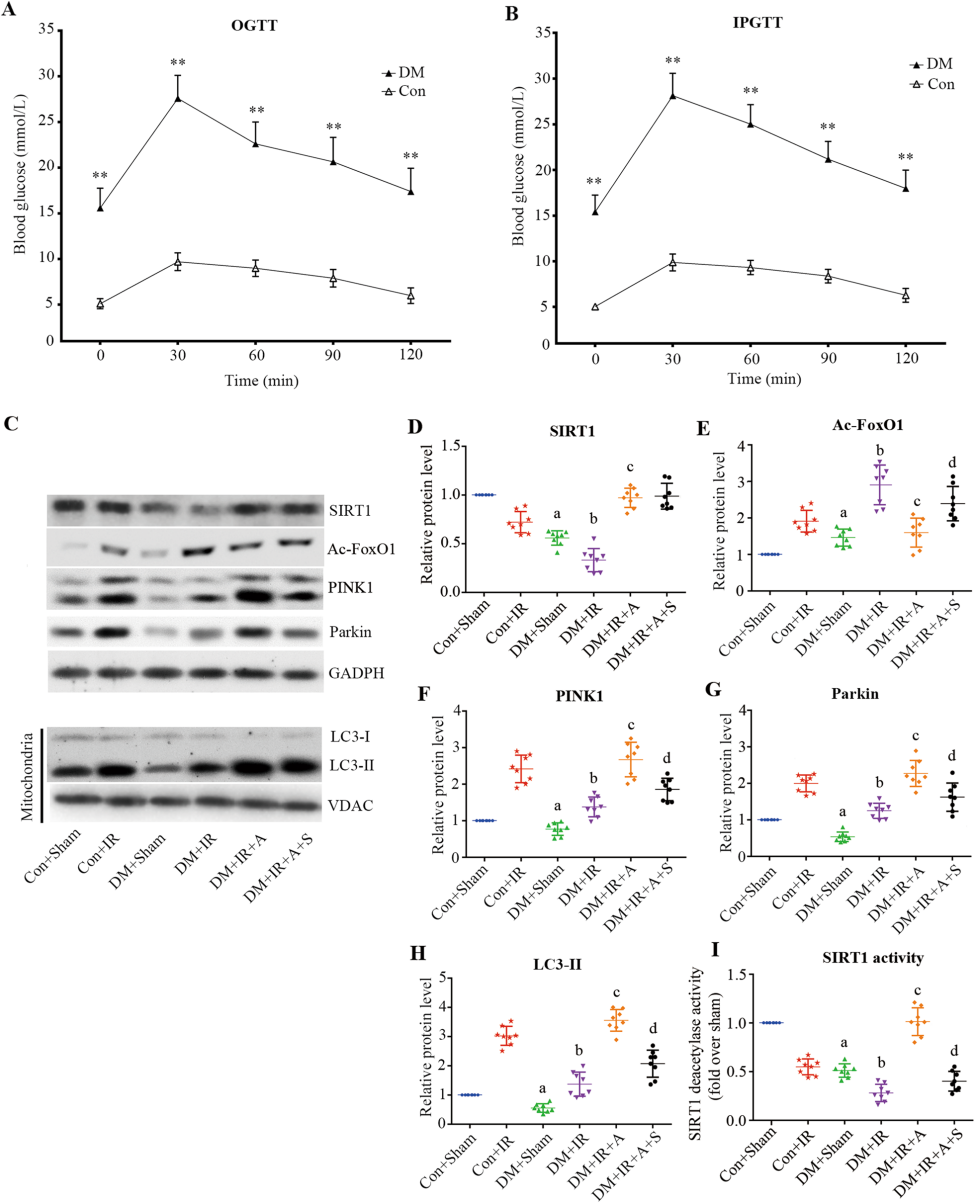
Diabetes reduced lung IR-induced mitophagy, and APN restored diabetic lung IR-reduced mitophagy via SIRT1. **A** OGTT, oral glucose tolerance test (mean ± SD). **B** IPGTT, intraperitoneal glucose tolerance test (mean ± SD). **C** Representative blots. **D** SIRT1 expression in lung tissue (mean ± SD). **E** Acetylated-FoxO1 expression in lung tissue (mean ± SD). **F** PINK1 expression in lung tissue (mean ± SD). **G** Parkin expression in lung tissue (mean ± SD). **H** LC3-II expression in mitochondria of lung tissue (mean ± SD). **I** Relative SIRT1 activity in lung tissue (mean ± SD). *Ac-FoxO1* acetylated Forkhead box O1, *LC3-II* microtubule-associated protein 1 light chain 3 beta, *PINK1* PTEN-induced kinase. (^a^*P* < 0.05 versus the Con + Sham group, ^b^*P* < 0.05 versus the Con + IR group, ^c^*P* < 0.05 versus the DM + IR group, ^d^*P* < 0.05 versus the DM + IR + A group; n = 8 in each group)

As shown in Fig. 1A, B, compared with non-diabetic rats, diabetic rats showed significantly impaired OGTT and IPGTT (*P* < 0.05), demonstrating that the type 2 diabetic model was successfully developed.

### Diabetes reduces lung IR-induced mitophagy, and APN restores diabetic lung IR-reduced mitophagy via SIRT1

We have reported that impaired lung SIRT1 signaling associated with type 2 diabetic conditions was further attenuated by IR injury in a warm lung IR model [17]. Similar tendencies were also observed in the diabetic lung transplantation model (Fig. 1C, I). We also found that adiponectin (APN) treatment restored the expression and activity of SIRT1 (*P* < 0.0001). Furthermore, EX527 treatment decreased SIRT1 activity and increased FoxO1 acetylation without influencing SIRT1 expression. (*P* < 0.0001, Fig. 1C, D and I).

The expression of mitophagy-related proteins was evaluated to assess the state of mitophagy. The conversion of LC3-I to LC3-II is a hallmark of autophagosome formation [34]. One paradigm for mitophagy involves the well-established PINK1 (PTEN-induced kinase)-Parkin pathway, which tags mitochondria for degradation [35]. As presented in Fig. 1C, the levels of LC3-II in the mitochondrial fraction and PINK1 and Parkin in the DM + Sham group were decreased (*P* < 0.05). The expression level of proteins was also attenuated in the DM + IR group compared with the Con + IR group (*P* < 0.0001), suggesting that mitophagy was suppressed in lung IR injury under type 2 diabetic conditions. Treatment with APN improved the levels of mito-LC3-II, which was accompanied by increases in PINK1 and Parkin (*P* < 0.05), however, inhibition of SIRT1 treatment attenuated these effects (*P* < 0.05).

### SIRT1 signaling pathway-mediated mitophagy participates in APN-mediated pulmonary protection of diabetic lung IR injury

**Fig. 2:**
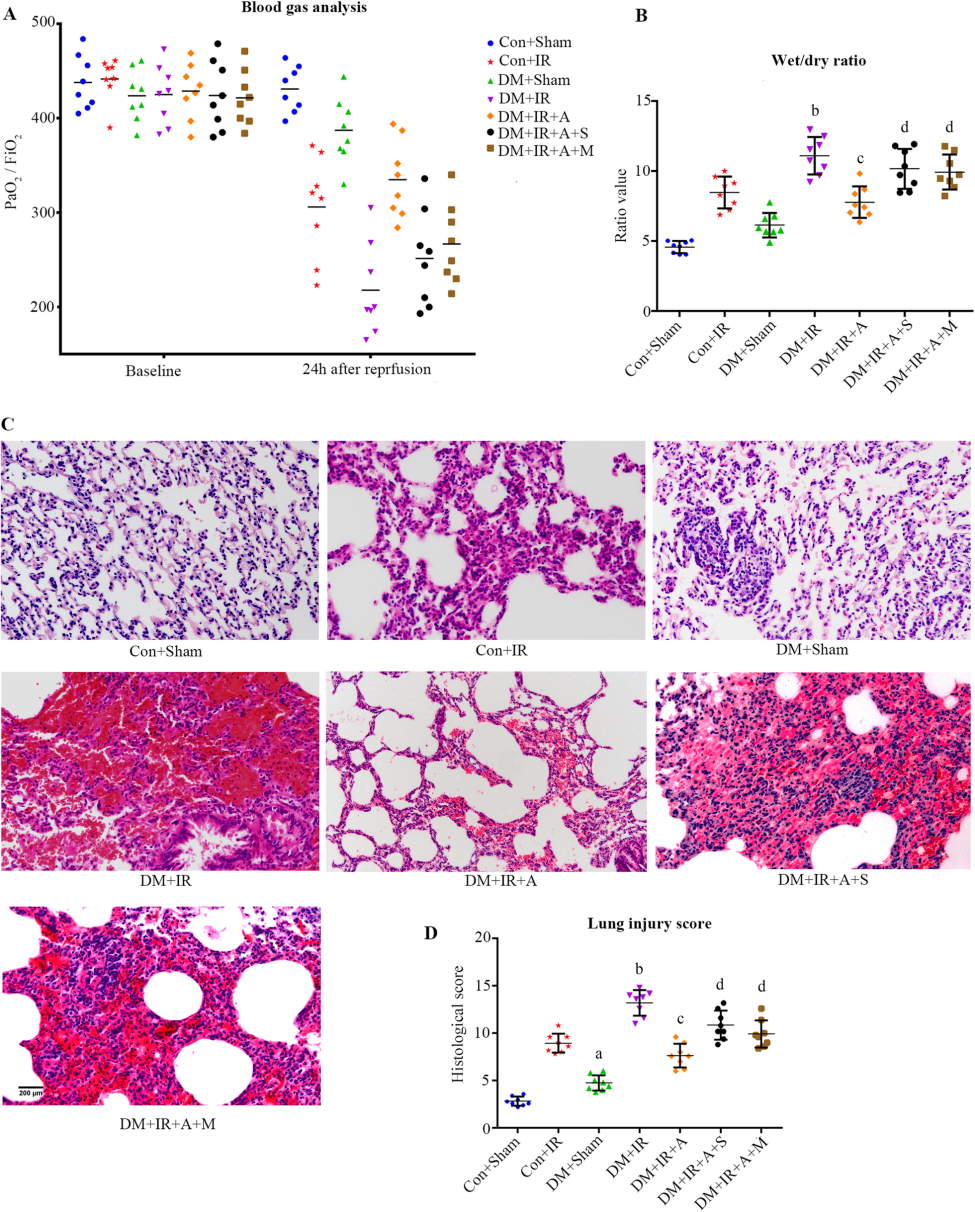
SIRT1 signaling pathway-mediated mitophagy participated in APN-mediated pulmonary protection in diabetic lung IR injury. **A** Arterial blood gas analysis (medians, interquartile range). **B** Wet/dry weight ratio (medians, interquartile range). **C** Histologic analysis of lung tissues (scale bars, 200 μm). **D** Lung injury score (medians, interquartile range). PaO_2_/FiO_2_, partial pressure of arterial oxygen (PaO_2_)/fraction of inspired oxygen (FiO_2_). (^a^*P* < 0.05 versus the Con + Sham group, ^b^*P* < 0.05 versus the Con + IR group, ^c^*P* < 0.05 versus the DM + IR group, ^d^*P* < 0.05 versus the DM + IR + A group; n = 8 in each group)

To confirm that the benefits of APN are closely related to mitophagy, the autophagy inhibitor 3-MA was used. As shown in Fig. 2A, The PaO_2_/FiO_2_ ratio in the Con + Sham group and the DM + Sham group showed no difference (*P* > 0.05). The PaO_2_/FiO_2_ ratio during reperfusion at 24 h was decreased in diabetic rats subjected to lung IR injury (*P* = 0.016). The wet weight-to-dry weight exhibited the opposite trend of the PaO_2_/FiO_2_ ratio. These data suggested hypoxemia and lung edema were aggravated in lung IR injury under type 2 diabetic conditions. APN administration significantly increased the PaO_2_/FiO_2_ ratio (*P* = 0.001). However, inhibition of SIRT1 abolished the effect of APN on the PaO_2_/FiO_2_ ratio (*P* = 0.0064), and 3-MA administration also attenuated the effect of APN on the PaO_2_/FiO_2_ ratio (*P* = 0.035). Increases in the wet weight-to-dry weight ratio were correlated with decreases in the PaO_2_/FiO_2_ ratio (Fig. 2B).

As presented in Fig. 2C, grafts in the Con + IR group showed lung edema, increased alveolar damage, hemorrhage, hyaline membrane formation, and inflammatory infiltrates. The lung injury scores in the DM + IR group were greater than those in the Con + IR group (*P* < 0.0001). These data suggested lung injury was aggravated in lung IR injury under type 2 diabetic conditions. APN treatment attenuated histologic changes (*P* < 0.0001), while SIRT1 inhibition aggravated histologic changes (*P* = 0.0004). The benefits of APN on histologic changes were also reversed by 3-MA (*P* = 0.0156).

### SIRT1 signaling pathway-mediated mitophagy plays an essential role in antiapoptotic, anti-inflammatory and antioxidative effects of APN following diabetic lung IR injury

**Fig. 3:**
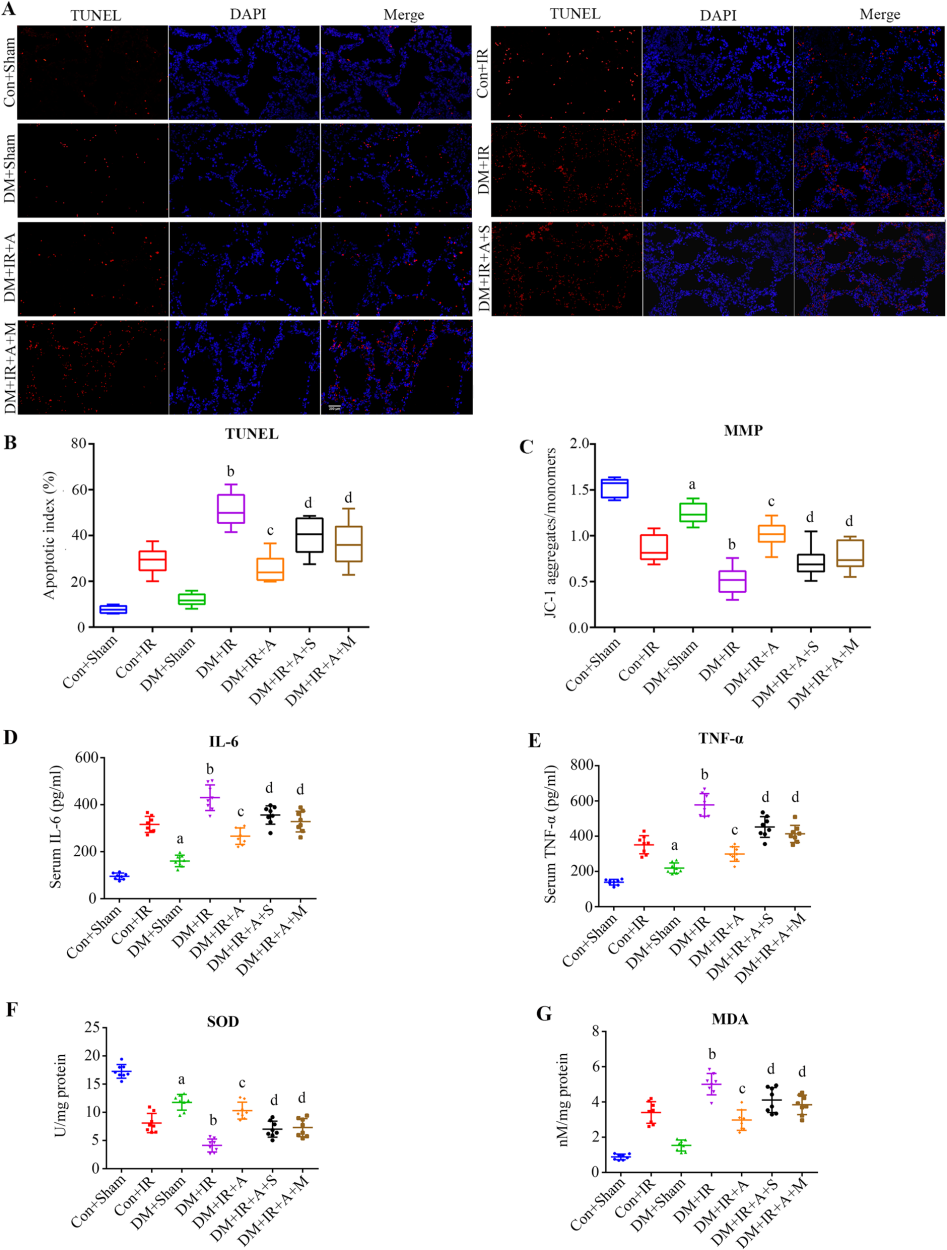
SIRT1 signaling pathway-mediated mitophagy playes an essential role in the antiapoptotic, anti-inflammatory and antioxidative effects of APN following diabetic lung IR injury. **A** Representative in situ detection of lung parenchymal cell apoptosis by TUNEL staining (scale bars, 200 μm). **B** Percentage of TUNEL-positive nuclei (medians, interquartile range). **C** Determination of mitochondrial membrane potential (medians, interquartile range). **D** Serum concentrations of interleukin-6 (IL-6) (mean ± SD). **E** Serum concentrations of TNF-α (mean ± SD). **F** lung concentrations of SOD (mean ± SD). **G** lung concentrations of MDA (mean ± SD). *IL* interleukin, *MDA* malondialdehyde, *MMP* mitochondrial membrane potential, *SOD* superoxide dismutase, *TNF-α* tumor necrosis factor-α, *TUNEL* terminal deoxynucleotidyl transferase dUTP nick end-labeling. (^a^*P* < 0.05 versus the Con + Sham group, ^b^*P* < 0.05 versus the Con + IR group, ^c^*P* < 0.05 versus the DM + IR group, ^d^*P* < 0.05 versus the DM + IR + A group; n = 8 in each group)

The apoptotic index of lung grafts was determined by TUNEL assay (Fig. 3A). The DM + IR group exhibited a higher apoptosis index than the Con + IR group (*P* < 0.0001). JC-1, a fluorescent dye highly sensitive to any small changes in mitochondrial membrane potential (Δψm), exhibited the opposite trend of the TUNEL assay (Fig. 3C). Serum concentrations of IL-6, and TNF-a, oxidative stress (MDA) showed the same trend as the apoptotic index, and the antioxidative capacity (SOD activities) exhibited the opposite trend compared with the previous levels (Fig. 3). These data suggested cell apoptosis, oxidative damage, and inflammation were aggravated in lung IR injury under type 2 diabetic conditions. The number of apoptotic cells in the lung grafts was significantly reduced in the DM + IR + A group (*P* < 0.0001), but the antiapoptotic effects of APN were mitigated in the DM + IR + A + S group (*P* = 0.0029). The antiapoptotic effect of APN was also abolished in the DM + IR + A + M group (*P* = 0.0235). Serum concentrations of IL-6, and TNF-a, oxidative stress (MDA) showed the same trend as the apoptotic index, and JC-1, the antioxidative capacity (SOD activities) exhibited the opposite trend compared with the previous levels (Fig. 3).

### Identification of PMVECs

**Fig. 4:**
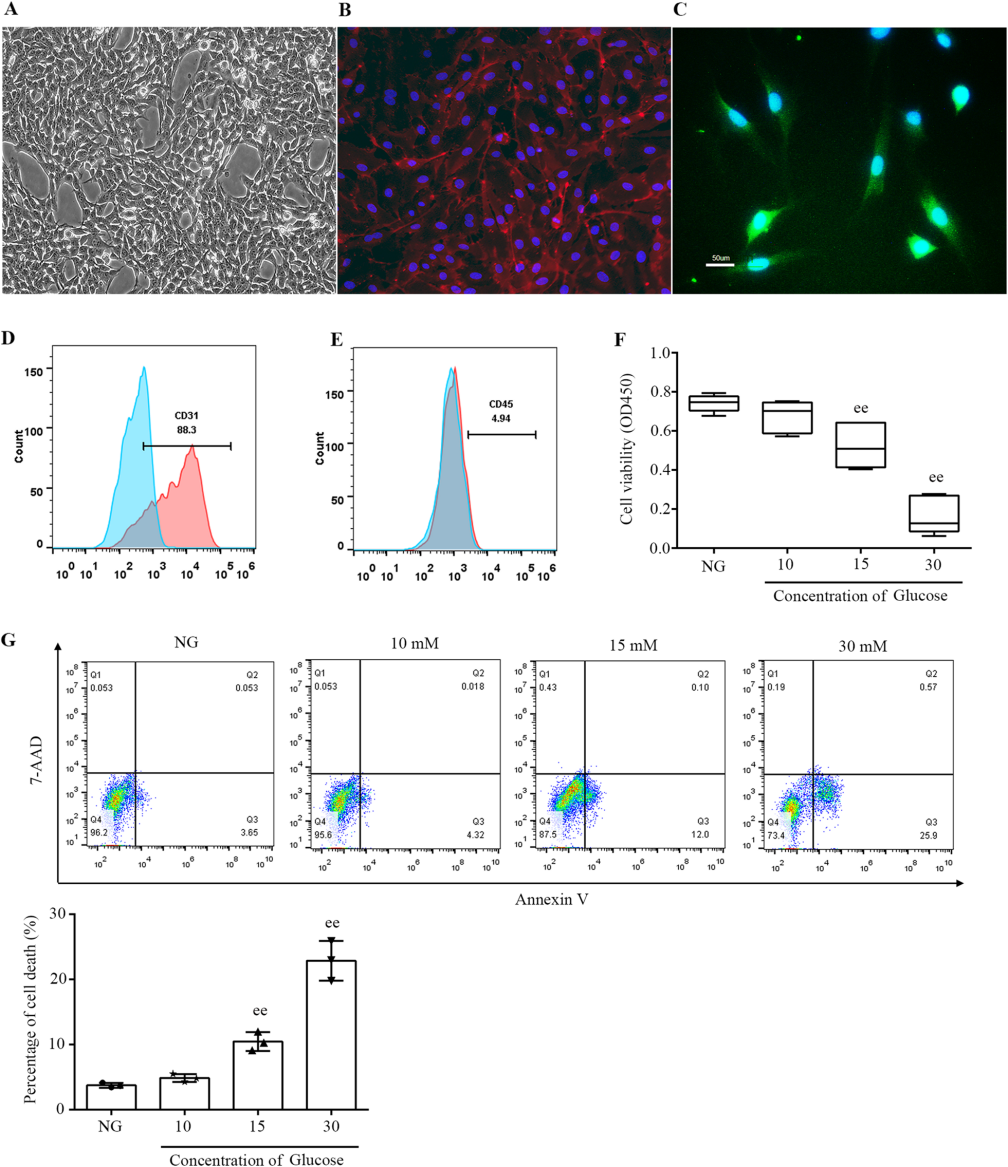
Characteristics of primary rat PMVECs. **A** Cobblestone appearance could be observed under an inverted microscope. **B** Cell nucleus DAPI staining merged with CD31 immunofluorescence staining. The nucleus showed blue fluorescence, and CD31 antigen expression showed red fluorescence in PMVECs. **C** Cell nucleus DAPI staining merged with FITC-conjugated lectin. The cytoplasm showed green fluorescence (scale bars, 50 μm). **D** The purity of PMVECs was about 88%. **E** PMVECs were deficient in CD45. **F** PMVECs-viability was measured by the CCK-8 kit (mean ± SD). **G** Cell apoptosis was determined through the flow cytometry in rat PMVECs (medians, interquartile range). ^ee^*P* < 0.05 versus the Control (NG)

To further elucidate the mechanisms underlying the action of APN on diabetic lung IR injury, we used PMVECs to establish an in vitro model of diabetic transplanted lungs [30]. Confluent PMVECs showed a cobblestone appearance and microvascular structures (Fig. 4A). The cells were identified by CD31 antigen and FITC-conjugated lectin expression, which are classical and highly specific markers of PMVECs (Fig. 4B, C). Flowcytometric analysis indicated that the purity of PMVECs was about 88% (Fig. 4D) [36]. PMVECs used in this study were negative for CD45 (Fig. 4E).

To explore favorable levels of glucose to mimic the pathophysiological condition of the diabetic state, PMVECs were exposed to glucose at final concentrations of 10, 15 and 30 mM with FFA palmitate (16:0; 500 μM) in cultures [31, 37, 38]. As shown in Fig. 4F, G, no effect was evident in the cultures exposed to 10 mM glucose for 24 h, whereas 30 mM glucose caused severe cell death. Therefore, 15 mM glucose was used in the present study because it may mimic the glucose levels in the diabetic state without insulin treatment and was also commonly used in previous studies [37].

### Alterations in mitochondrial ROS production, Δψm and mitophagy in PMVECs subjected to diabetic IR injury

**Fig. 5:**
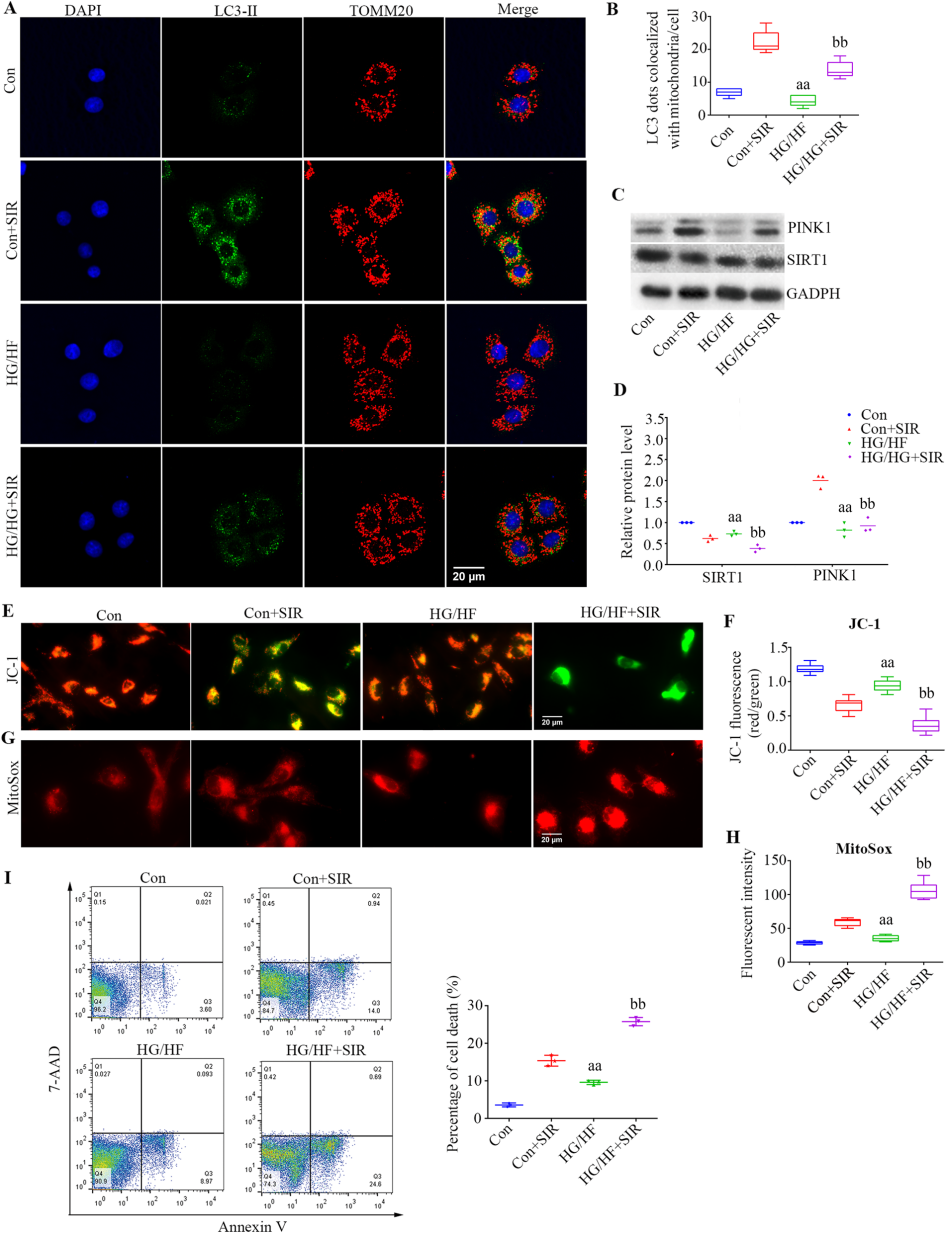
Alterations in mitochondrial ROS production, Δψm and mitophagy in PMVECs subjected to diabetic IR injury. **A** The coimmunofluorescence of LC3-II (fluorescent green) and TOMM20 (fluorescent red) was assessed to measure the level of mitophagy in PMVECs in each group (scale bars, 20 μm). **B** Quantitative analysis of mitophagosome formation (mean ± SD). **C** Representative blots. **D** SIRT1 and PINK1 expression (mean ± SD). **E** Mitochondrial membrane potential visualized by JC-1, a dye sensitive to mitochondrial membrane potential changes (scale bars, 20 μm). **F** Quantification of JC-1 fluorescence intensity (red/green florescent area) (mean ± SD). **G** The amount of ROS in PMVECs was determined by MitoSOX (scale bars, 20 μm). **H** Quantification of MitoSOX fluorescence intensity (red) (mean ± SD). **I** Cell apoptosis was determined by flow cytometry in rat PMVECs (medians, interquartile range). *HF* high fat, *HG* high glucose, *SIR* simulated ischemia–reperfusion. (^aa^*P* < 0.05 versus the Con group, ^bb^*P* < 0.05 versus the Con + SIR group)

LC3-II staining was used to identify for autophagosomes, TOMM20 was used as marker for mitochondria, and a combination of LC3-II and TOMM20 was used to delineate mitophagy. The extent of mitophagy was examined by the number of LC3-II puncta on mitochondria per cell. As presented in Fig. 5A, mitophagy was decreased in the HG/HF group compared with the Con group (*P* = 0.01), and mitophagy was also decreased in the HG/HF + SIR group (*P* < 0.0001). These data further confirmed that HG/HF-inhibited mitophagy in PMVECs subjected to IR injury. As presented in Fig. 5C, the levels of SIRT1 and PINK1 were decreased (*P* < 0.05, the HG/HF group compared with the Con group). The expression levels of proteins were also attenuated in the HG/HF + SIR group compared with the Con + SIR group (*P* < 0.05). Mitochondrial dysfunction and accumulation of damaged mitochondria may contribute to ROS generation and cell death. We measured the levels of mitochondrial ROS in PMVECs by MitoSOX (red). As shown in Fig. 5G, mitochondrial ROS levels were increased in the HG/HF group (*P* = 0.0143), and the levels were increased in the HG/HF + SIR group (*P* < 0.0001). HG/HF also induced mitochondrial depolarization following IR injury, as evidenced by diffuse JC-1 green staining (*P* < 0.05, Fig. 5E). The rate of PMVECs apoptosis showed the same trend as mitochondrial ROS levels (Fig. 5I).

### APN upregulated PINK1-dependent mitophagy via the SIRT1 signaling pathway in PMVECs subjected to diabetic IR injury

**Fig. 6:**
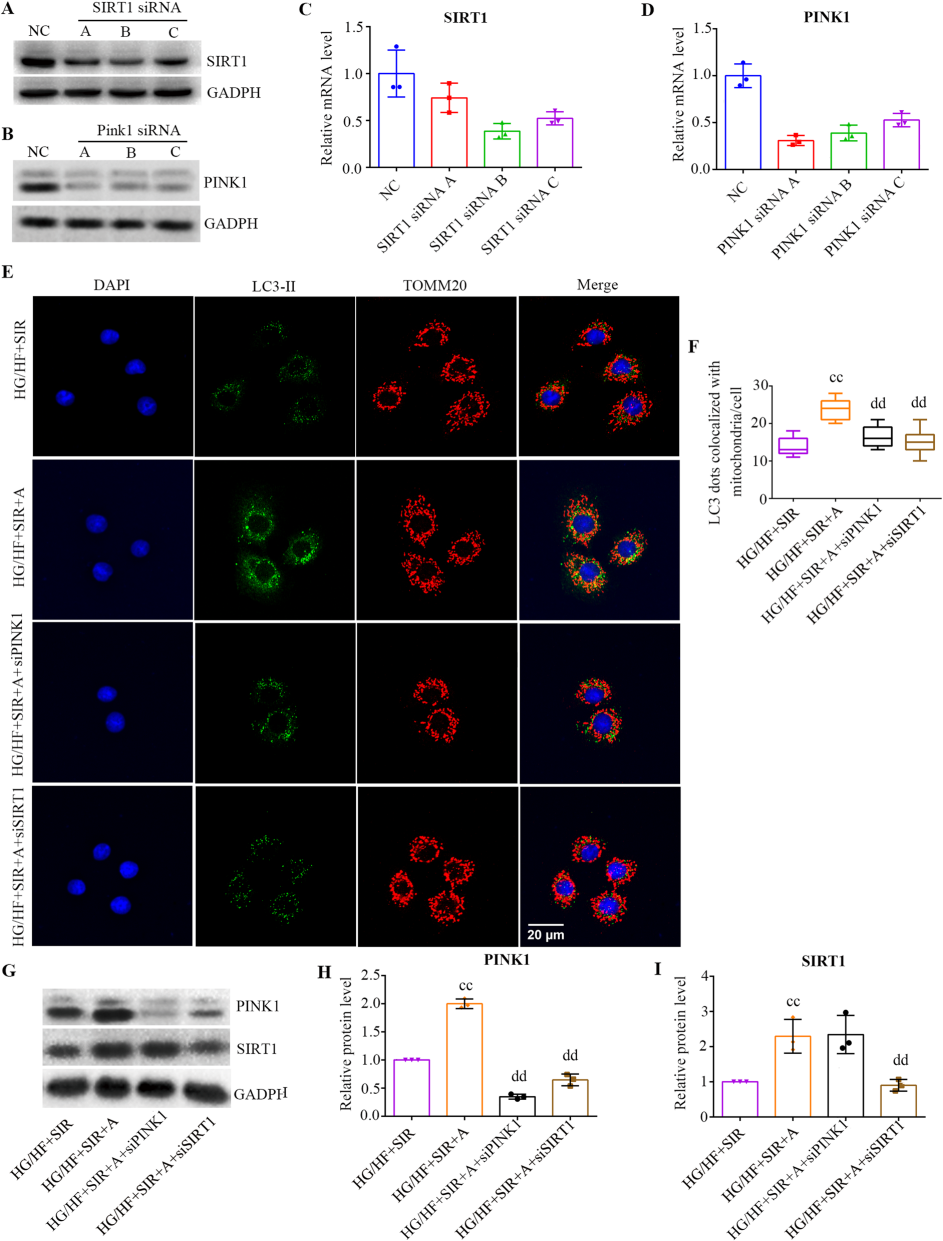
APN upregulated PINK1-dependent mitophagy via the SIRT1 signaling pathway in PMVECs subjected to diabetic IR injury. **A** PMVECs were infected with SIRT1 siRNA (**A**–**C**) and a negative control (NC), and representative blots are shown. **B** PMVECs were infected with PINK1 siRNA (**A**–**C**) and a negative control (NC), and representative blots. **C** The mRNA expression of SIRT1 (mean ± SD). **D** The mRNA expression of PINK1 (mean ± SD). **E** The coimmunofluorescence of LC3-II (fluorescent green) and TOMM20 (fluorescent red) was assessed to measure the level of mitophagy in PMVECs in each group (scale bars, 20 μm). **F** Quantitative analysis of mitophagosome formation (mean ± SD). **G** Representative blots. **H** PINK1 expression (mean ± SD). **I** SIRT1 expression (mean ± SD). HF, high fat; HG, high glucose; SIR, simulated ischemia–reperfusion. (^cc^*P* < 0.05 versus the HG/HG + SIR group, ^dd^*P* < 0.05 versus the HG/HG + SIR + A group)

Next, we explored some insights into the regulation of mitophagy in our experimental models, and PMVECs infected with *PINK1* siRNA or *SIRT1* siRNA. The expression of *SIRT1* mRNA and protein was dramatically reduced by all 3 *SIRT1* siRNAs compared with that in the negative control group, with B being more effective than A and C (Fig. 6A, C). Similar changes were observed with *PINK1* siRNA, with A being more effective than B and C (Fig. 6B, D). We therefore selected *SIRT1* siRNA B, and *PINK1* siRNA A for further analysis.

As shown in Fig. 6E, APN treatment significantly enhanced mitophagy in PMVECs exposed to diabetic IR injury compared with that observed in untreated PMVECs (*P* < 0.0001). Having demonstrated that APN significantly increased mitophagy, we next determined the molecular mechanisms mediating this effect in diabetic IR injury. Western blot assays showed that APN treatment notably enhanced the expression levels of PINK1 in PMVECs exposed to diabetic IR injury (*P* < 0.0001, Fig. 6G). As shown in Fig. 6E, gene silencing of PINK1 markedly abolished the effect of APN on the upregulation of mitophagy, suggesting that the PINK1 pathway plays a central role in the action of APN on mitophagy. We next investigated the mechanism underlying the induction of PINK1-dependent mitophagy by APN, and the SIRT1 signaling pathway was evaluated because previous studies have identified SIRT1 as the upstream mediator of PINK1 [39]. As shown Fig. 6G, western blotting analysis demonstrated that both SIRT1 and PINK1 were repressed by diabetic IR injury, and APN treatment upregulated the levels of SIRT1 as well as PINK1 in PMVECs (*P* < 0.05). Gene silencing of SIRT1 caused a decline in PINK1 expression (*P* = 0.0016), whereas gene silencing of PINK1 had little effect on SIRT1 expression (*P* > 0.05), suggesting that PINK1 expression was modulated by the SIRT1 signaling pathway. Interestingly, we also found that SIRT1 gene silencing markedly abolished the effect of APN on upregulation of mitophagy (*P* < 0.05, Fig. 6E).

### APN conferred protective effects on mitochondrial function through SIRT1- PINK1-dependent mitophagy in PMVECs subjected to diabetic IR injury

**Fig. 7:**
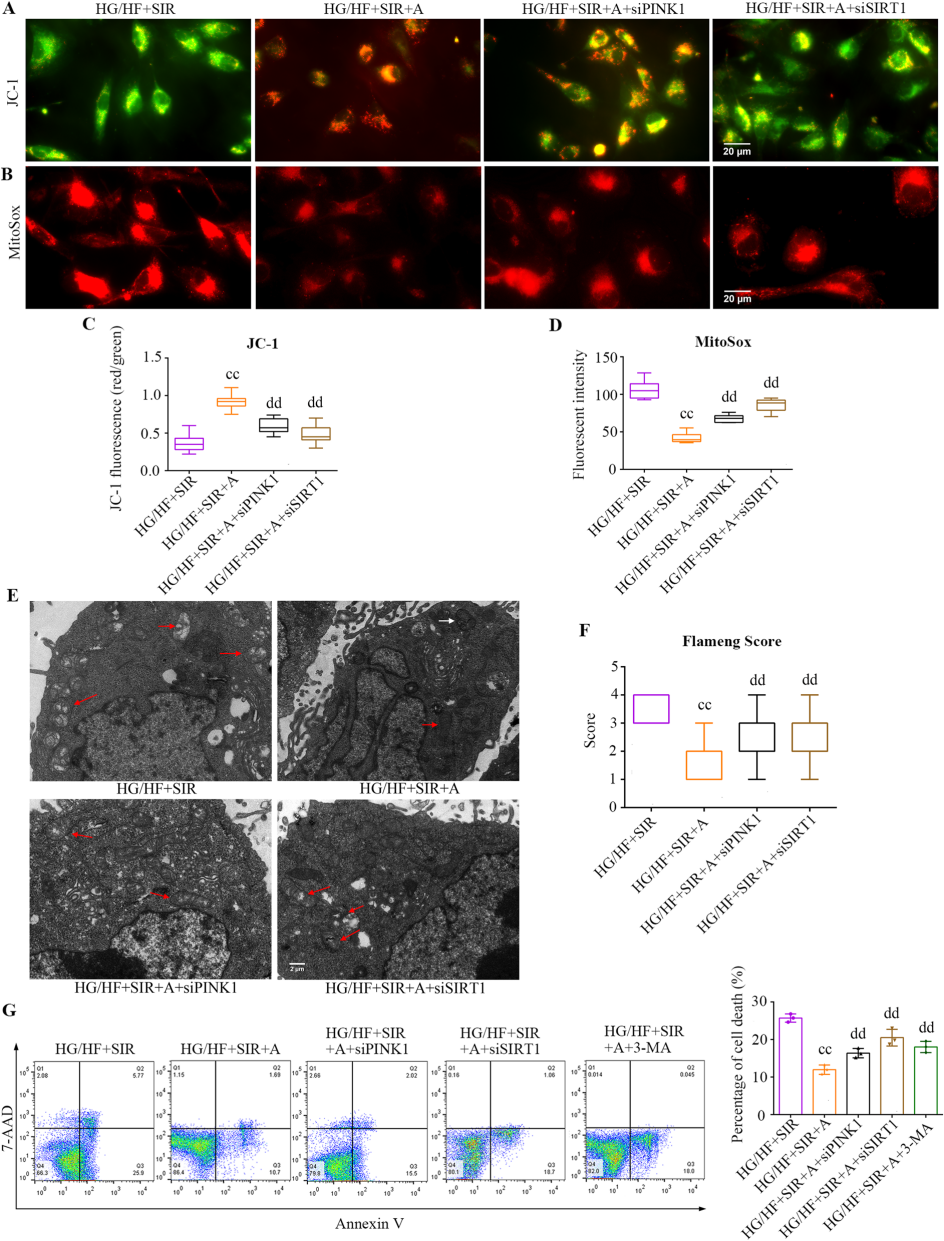
APN conferred protective effects through SIRT1- PINK1-dependent mitophagy in PMVECs subjected to diabetic IR injury. **A** Mitochondrial membrane potential visualized by JC-1(scale bars, 20 μm). **B** The amount of ROS in PMVECs was determined by MitoSOX (scale bars, 20 μm). **C** Quantification of JC-1 fluorescence intensity (red/green florescent area) (mean ± SD). **D** Quantification of MitoSOX fluorescence intensity (red) (mean ± SD). **E** Mitochondrial ultrastructure. Mitochondria (red arrows) and mitophagosome (white arrows) in PMVECs were imaged with transmission electron microscopy (scale bars, 2 μm). **F** Flameng score (medians, interquartile range). **G** Cell apoptosis was determined by flow cytometry in rat PMVECs (medians, interquartile range). *HF* high fat, *HG* high glucose, *SIR* simulated ischemia–reperfusion. (^cc^*P* < 0.05 versus the HG/HG + SIR group, ^dd^*P* < 0.05 versus the HG/HG + SIR + A group)

We next examined the effects of mitophagy on APN-mediated cytoprotection in PMVECs subjected to IR injury. As shown in Fig. 7A, APN treatment restored Δψm, allowing JC-1 uptake by mitochondria (red staining) (*P* < 0.0001), while either knocking down SIRT1 or PINK1 expression attenuated the effect of APN on mitochondrial JC-1 uptake (*P* < 0.0001). As shown in Fig. 7B, APN treatment reduced mitochondrial ROS (*P* < 0.0001), while knocking down SIRT1 expression attenuated the effect of APN on mitochondrial ROS (*P* < 0.0001), knockdown of PINK1 expression also mitigated the effect of APN on mitochondrial ROS (*P* < 0.0001). Next, we examined the mitochondrial morphological changes in PMVECs under transmission electron microscope. As shown in Fig. 7E, the PMVECs in the HG/HF + SIR group exhibited mitochondrial edema, cristae rupture, matrix concentration and mitochondrial membrane rupture. APN treatment attenuated mitochondrial morphological changes, and the Flameng scores in the HG/HF + SIR + A group were lower than those in the HG/HF + SIR group (*P* < 0.0001). Mitophagosome formation was also found in the HG/HF + SIR + A group. However, knocking down PINK1 expression attenuated the effect of APN on mitochondrial morphological changes (*P* = 0.0288), and knocking down SIRT1 expression also mitigated the effect of APN on mitochondrial morphological changes (*P* = 0.0023). To investigate the beneficial action of APN-modulated mitophagy on PMVECs, 3-MA was administered after APN-treatment. As showed in Fig. 7G, APN notably reduced the rate of PMVECs apoptosis caused by diabetic IR injury (*P* < 0.0001), whereas knockdown of either SIRT1 or PINK1 expression abolished the effect of APN (*P* < 0.05). Of note, the benefits of APN were closely related to mitophagy as the rescue effects of APN were reversed by 3-MA treatment (*P* = 0.0004).

## Discussion

The involvement and modulation of mitophagy in the pathogenesis of diabetic lung IR injury remain poorly understood. The data presented in this study reflect several important observations. First, our study demonstrated that DM aggravated lung IR injury during lung transplantation in vivo and in vitro. Second, our results indicated that type 2 diabetes severely impaired mitophagy following lung transplantation. Third, we found evidence that, by upregulating SIRT1- PINK1-dependent mitophagy, APN might ameliorate mitochondrial dysfunction, thus reducing ROS production and inflammation, contributing to cell survival and ultimately preserving lung function during diabetic lung IR injury.

The incidence of diabetes continues to increase worldwide, and patients with pretransplant DM have poor survival compared with the nondiabetic population of lung transplantation recipients [7]. In this study, we developed a new diabetic lung transplantation model by using type 2 diabetic rats as recipients, which simulated the events associated with clinical lung transplantation recipients with pretransplant DM. Consistent with our previous report on a warm diabetic lung IR model, we found that DM aggravated lung IR injury in the transplantation model [9, 17].

Hyperglycemia leads to overproduction of reactive oxygen species that then potentially provoking mitochondrial dysfunction, and damaged mitochondria may predispose cells to free radical generation and eventual cell death [40]. ROS and various factors released upon cell death potentially provoke inflammation [41]. In response to dysfunctional mitochondria, mitochondrial quality control mechanisms can be activated to allow autophagosomes to selectively eliminate impaired mitochondria, which is known as mitophagy [11]. The best-characterized mitophagy pathway to date is the PINK1-Parkin pathway [42]. Mitochondrial dysfunction is a critical factor in lung IR injury [43]. Increasing lines of evidence have demonstrated a protective role of PINK1-Parkin mediated mitophagy in IR injury [26]. In the current study, we detected the induction of PINK1-Parkin mediated mitophagy in both in vitro and in vivo models of lung IR injury. Of note, convincing evidence has shown that mitophagy through PINK1-Parkin degradation mechanisms is suppressed in type 2 DM, which contributes to the accumulation of dysfunctional mitochondria [44, 45]. Conflicting observations have been reported in mice fed with high-fat diet, Tong et al. observed that mitophagy activated during the initial phase of high-fat diet consumption was both Atg7-and Parkin-dependent in cardiomyocytes. However, Tong et al. also found that mitophagy was activated but insufficient for the maintenance of mitochondrial function during the early phase of diabetic cardiomyopathy [46]. Our current study demonstrated that mitophagy is suppressed in both high-fat diet-fed streptozotocin induced type 2 diabetic rats and in high-glucose, high-fat medium in vitro. The mitophagy detected in this study was different from that observed in the work of Tong, which is most likely due to the different experimental models. However, a common finding in different models and in clinical settings highlights the importance of mitophagy in diabetic complications. Δψm is a global index of mitochondrial function. PINK1 deficiency under diabetic conditions enhanced Drp1-dependent mitochondrial fragmentation, which leaded to mitochondrial membrane depolarization [47]. Surveillance of mitochondrial quality control allows these injured organelles to be recycled by mitophagy [10]. We presented data showing that mitophagy was suppressed under diabetic conditions suggesting compromised degradation of damaged mitochondria and a less efficient surveillance mechanism, which induced excessive ROS generation, and ultimately resulted in Δψm depolarization and initiation of the mitochondrial apoptotic cascade (Fig. 5). ROS and various factors released upon cell death might also be potent activators of inflammation. All these factors might play a pivotal role in the development and progression of diabetes and its complications. Our previous studies have shown that mitochondrial dysfunction caused by DM leads to aggravated lung IR injury [9, 17], and the resultant impaired mitochondria increase cell’ susceptibility to free radical generation and eventual cell apoptosis. One contributing factor in diabetic lung IR injury is the excessive ROS generation, which can damage the integrity of the lung epithelia and disrupt the alveolar-capillary interaction, contributing to lung edema [9]. Here, we presented evidence that APN can preserve lung function and prevent lung edema in diabetic lung IR injury, which is consistent with our previous report [21]. The data presented in the current study also showed that mitophagy was suppressed under diabetic conditions, which was accompanied by mitochondrial membrane depolarization, and increased mitochondrial ROS, mitochondrial damage, inflammation and cell apoptosis during lung IR injury. Therefore, this study implied a relationship between the mechanisms underlying the diabetic state aggravating lung IR injury and impaired mitophagy.

SIRT1 has been proposed to maintain mitochondrial homeostasis by regulating ROS, mitophagy and mitochondrial biogenesis [48]. Recent studies have highlighted the importance of SIRT1 on the treatment of IR injury [15]. We recently assessed the role of SIRT1 in H_2_S’s protective effect against lung IR injury in type 2 diabetic rats [17]. APN has been well demonstrated to exert a modulatory effect on mitochondrial biogenesis and mitophagy [20, 49]. Of interest, David et al. observed that APN protected against IR injury via SIRT1 signaling [50]. Thus, we heavily focused on the relationship between APN and SIRT1. Consistent with our previous report on a warm diabetic lung IR model, the data presented in this study showed that lung SIRT1 signaling was dramatically downregulated under type 2 diabetic conditions, and it was further attenuated by ischemia–reperfusion injury accompanied by impaired mitophagy during diabetic lung transplantation. Our study further found that through enhanced clearance of damaged mitochondria via mitophagy, APN suppressed mitochondrial depolarization, reduced mitochondrial ROS generation, attenuated mitochondrial injury, decreased inflammation, and diminished apoptosis. Accumulating evidence suggests that SIRT1 induces mitophagy, contributing to attenuation of mitochondrial dysfunction and subsequent IR injury [15]. SIRT1 modulates the expression of FoxO1, which promotes mitophagy via the PINK1-Parkin pathway to protect against mitochondrial dysfunction under diabetic conditions [51]. SIRT1-FoxO1 signaling maintains mitochondrial homeostasis by mediating mitophagy [52]. In the current study, we found a change in the PINK1-dependent mitophagy signaling pathway with or without administration of APN, and demonstrated that APN treatment induced pathological improvements accompanied by upregulation of PINK1-dependent mitophagy during diabetic lung IR injury. We found that APN restored FoxO1 deacetylation by SIRT1. As SIRT1 acts upstream of PINK1, we also explored mechanisms underlying the effects of APN on upregulating mitophagy under diabetic lung IR conditions. Our study demonstrated that SIRT1, activated by APN, contributed to PINK1-dependent mitophagy, eventually inducing pro-survival signals for the reperfused diabetic lung and mitochondria. However, the molecular motifs that directly control the SIRT1- PINK1 interaction during diabetic lung IR remain to be evaluated.

Considering the high incidence of DM after lung transplantation, which is generally assumed to be of new onset as treatment involves high dose glucocorticoid therapy to prevent rejection [8], attenuation of attenuate diabetic lung IR injury with APN treatment may contribute to the development of a novel therapeutic strategy for diabetic patients following lung transplantation. Adiponectin, as a modulator of immunity and inflammation, adiponectin might provide a new therapeutic target for allograft rejection after lung transplantation [53, 54]. Accumulating evidence suggests that mitophagy may restrict inflammatory cytokine secretion and regulate mitochondrial antigen presentation and immune cell homeostasis [55]. In the absence of PINK1-Parkin dependent mitophagy, mitochondrial stress can lead to the release of mtDNA triggering TMEM173-dependent production of cytokines, such as IL-6 [56]. Our study showed that APN suppressed proinflammatory factors (TNF-α and IL-6) through SIRT1- PINK1 signaling-mediated mitophagy, leading to inhibition of lung IR injury, which might mitigate the subsequent occurrence of acute graft rejection and obliterative bronchiolitis [6, 57]. Additional studies need to elucidate the mechanisms by which APN regulates the immune system through mitophagy to advance our knowledge of APN translational applications in diabetic lung IR injury.

The current study raised additional questions that we have yet to resolve. First, we did not measure APN levels in plasma. Hypoadiponectinemia caused by diabetes is correlated with an increased risk of IR injury [58]. Second, since APN can exert a modulatory effect on autophagic flux, we cannot formally exclude the possibility that increases in general autophagy may also contribute to the protective effect of APN during diabetic lung IR injury. In view of the fact that Wang et al. demonstrated APN restored diabetes-induced autophagic flux arrest, thus ameliorating diabetic IR injury [20]. Third, although we showed that mitophagy was reduced during diabetic lung IR injury, how mitophagy influences mitochondrial biogenesis under these conditions remains unknown. Given that both APN and SIRT1 contribute to mitochondrial biogenesis, additional studies are needed to elucidate the potential roles of the mitochondrial biogenesis pathways in the APN treatment in diabetic lung IR injury. Fourth, we developed a high-glucose, high-fat medium-induced type 2 diabetic model in vitro and a high-fat diet-fed streptozotocin-induced type 2 diabetic model in vivo to mimic the pathophysiological condition of type 2 DM patients [31], but whether these models completely simulate for human diabetes requires further exploration. Furthermore, whether similar results could be obtained with female rats needs to be explored.

## Conclusion

In summary, our findings demonstrate that mitophagy is specifically increased by APN during diabetic lung IR injury, resulting in selective removal of damaged mitochondria, preservation of mitochondrial function, and inhibition of ROS, inflammation and apoptosis. The potential mechanisms of the roles of APN might involve upregulation of mitophagy through activation of SIRT1- PINK1 pathway.

## Data Availability

The datasets supporting the conclusions of this article are included within the article.

## Abbreviations

3-MA: 3-Methyladenine
APN: Adiponectin
DM: Diabetes mellitus
IR: Ischemia reperfusion
LC3-II: Microtubule associated protein 1 light chain 3 beta
PINK1: PTEN induced putative kinase 1
ROS: Reactive oxygen species
SIRT1: Sirtuin 1
siRNA: Small interfering RNA
TOMM20: Translocase of outer mitochondrial membrane 20 homolog (yeast
